# *In situ* real-time dataset as compared to the predicted thermal wheel performance of an energy recovery system

**DOI:** 10.1016/j.dib.2023.109472

**Published:** 2023-08-03

**Authors:** Hung-Yi Tsai, Chung-Tai Wu

**Affiliations:** Lee-Ming Institute of Technology, Taiwan

**Keywords:** Desiccant wheel, Moisture removal capacity (MRC), Process air, Regeneration air

## Abstract

The data article refers to *in situ* real-time dataset of a rotary desiccant wheel system and description of its experimental design. The original research paper was published in *Heliyon*, “Optimization of a rotary desiccant wheel for enthalpy recovery of air-conditioning in a humid hospitality environment” [1]. Supplementing to this data article are eight dataset files pertaining to ambient conditions and dehumidified process air outputs, as well as comparisons of experimental and numerical outputs under various operating conditions. Physical implementation of the experiments for numerical validation of transient responses included step changes to the regeneration air temperature, wheel speed, and airflow rate (i.e. fan speed). Finally, the data article provides insights to optimal performance of thermal wheel operation for HVAC practitioners and academic researchers.

**Specifications Table**

**Table:** 

Subject	Engineering
Specific subject area	Building energy recovery
Type of data	Tables (*.txt)
How data were acquired	Type-T thermocouples were used for temperature measurements. Chilled mirror hygrometers (D2 sensors) were used for humidity measurements. Data were obtained *in situ* via a plug-in HP 44421A guarded data acquisition board and stored in a personal computer.
Data format	Raw (labeled text files)
Description of data collection	The dataset includes *in situ* sensor-recorded experiments from 2018 to 2019, as well as simulated outputs for comparison. Step increase and step decrease were applied to the regeneration air temperature, wheel speed, and fan (air) speed to assess the respective transient effect of the process air output (dehumidified airstream) before air-conditioning.
Data source location	City/Town/Region: Taoyuan City, Taiwan. Primary data source site: Taoyuan Hotel Latitude and Longitude: 24^o^59′08”N, 121^o^18′13”E
Data accessibility	Repository name: Mendeley Data Data identification number: 10.17632/pwm6cg2shj.2 Direct URL to data: https://data.mendeley.com/datasets/pwm6cg2shj/2
Related research article	Tsai, H.Y., & Wu, C.T. (2022). Optimization of a rotary desiccant wheel for enthalpy recovery of air-conditioning in a humid hospitality environment. *Heliyon*, 8(10), e10796, ISSN 2405-8440, http://doi.org/10.1016/j.heliyon.2022.e10796.

## Value of the Data

• The dataset is valuable for the renewable energy research community of buildings in thermal comfort, HVAC control strategy, energy efficiency, sustainability, and similar others. The data allows engineers to design smart buildings for reduced energy consumption and carbon emissions.
• The dataset provides quantitative information on the design requirements of a rotary desiccant wheel system in buildings that require large cooling loads for energy recovery.
• The simulation data validated optimal control strategy of a rotary desiccant wheel under various parametric conditions: ambient differences, material properties, operational factors, and wheel geometry. A range of values was identified for optimal control strategy of the existing thermal wheel, as represented by the publication of Tsai and Wu [1].
• The dataset gives insight into the implementation of thermal wheels in buildings of hot and humid climates. Additionally, for any space that requires super low humidity such as semiconductor laboratory or medical surgery room, the utility of this dataset may also be applicable.

## 1 Objective

Optimization of a rotary desiccant wheel is a rigorous and time-consuming process, taking into account of varying ambient conditions (e.g. temperature, humidity) and operating conditions (e.g. wheel speed, airflow rate). This data article supplements the published research article, “Optimization of a rotary desiccant wheel for enthalpy recovery of air-conditioning in a humid hospitality environment” by Tsai and Wu [1], with complete data sets of experimental ambient temperature, ambient humidity, processed air temperature, and processed air humidity, as well as numerically generated process air temperature, process air humidity, and moisture removal capacity (MRC). The paper describes how the experimental work was conducted within an air-handling-unit (AHU) in real-time. Finally, validation of experimental and numerical data was achieved by these data sets for the completion of the published article in *Heliyon*.

## 2 Data Description

This paper presents experimental data of the desiccant wheel facility in Taoyuan Hotel, Taiwan. The dataset files of dataset1.txt, dataset2.txt, dataset3.txt, and dataset4.txt were recorded *in situ* real-time from 2018 to 2019 before the COVID pandemic halted the building operation. The dataset files of dataset5.txt, dataset6.txt, and dataset7.txt were simulated results as a comparison to the real-time data. Experimental results of dataset8.txt were not time-specific but for numerical validation of transient responses from step changes to the peripheral operating conditions. Brief description of each dataset is as follows:

*Dataset 1*: Ambient air dry-bulb temperatures (°C) before processing.
*Dataset 2*: Ambient air humidity (non-dimensional) before processing.
*Dataset 3*: Process air dry-bulb temperatures (°C) exiting the desiccant wheel, *blank* when wheel is not running (i.e. cooling operation is off during winter).
*Dataset 4*: Process air humidity (non-dimensional) exiting the desiccant wheel, *blank* when cooling operation is off.
*Dataset 5*: Simulated process air dry-bulb temperatures (°C) exiting the desiccant wheel, *blank* when cooling operation is off.
*Dataset 6*: Simulated process air humidity (non-dimensional) exiting the desiccant wheel, *blank* when cooling operation is off.
*Dataset 7*: Moisture removal capacity (kilogram of water removed per hour) of process air exiting the desiccant wheel per simulation, *blank* when cooling operation is off.
*Dataset 8*: Experimental and numerical comparisons of transient response due to step changes to the regeneration air temperature, wheel speed, and process airflow rate.

Generally, transient effects due to parametric changes would vary greatly. For example, the transient effect of the process air temperature output would fluctuate more sinuously with step changes of the airflow rate (i.e. increased or decreased fan speed) until reaching steady-state. Although the resulting transient effect of the process air temperature was also sinusoidal from step changes of the regeneration air temperature, the time it takes to reach steady-state was much longer. Moreover, the transient effect of the process air temperature output tends to oscillate due to step changes of the wheel speed. An illustration of the aforementioned phenomenon is shown in Table 1 where the shortest transient time to reach steady-state occurs with low regeneration air temperature, low wheel speed (9 revolutions per hour, rph), and high fan speed (800 feet per minute, fpm). On the other hand, it would take much longer to reach steady-state when the regeneration air temperature is high, coupled with low fan speed and high wheel speed. Based on conventional engineering practice that accepts three time-constants as steady-state, the paper defines steady-state when 95% of the average steady-state value is reached (as opposed to 4 time-constants that require 98%, 5 time-constants that require 99%).

**Table 1:** Step changes of regeneration air temperature, wheel speed, and fan speed.

	Initial state		Step change			Response reaching steady-state		
	Processed air inlet		Regeneration air	Wheel speed	Fan speed	Processed air outlet		
Run	*T* [°C]	*w* [kg/kg]	*T* [°C]	[rph]	[fpm]	*T* [°C]	*w* [kg/kg]	Transient time
1	32.4	.0183	68.8	18	400	59.3	.0119	16 min
2	33.1	.0186	68.5	18	800	59.5	.0115	13 min
3	32.7	.0179	68.1	9	800	58.8	.0126	7 min
4	32.5	.0177	67.7	9	400	58.4	.0118	12 min
5	33.8	.0219	79.3	9	400	61.2	.0115	24 min
6	33.3	.0195	79.6	9	800	61.7	.0117	21 min
7	33.6	.0192	51.2	18	800	47.1	.0122	5 min
8	33.9	.0221	51.5	18	400	48.6	.0124	10 min

## 3 Experimental Design, Materials and Methods

**Fig. 1:**
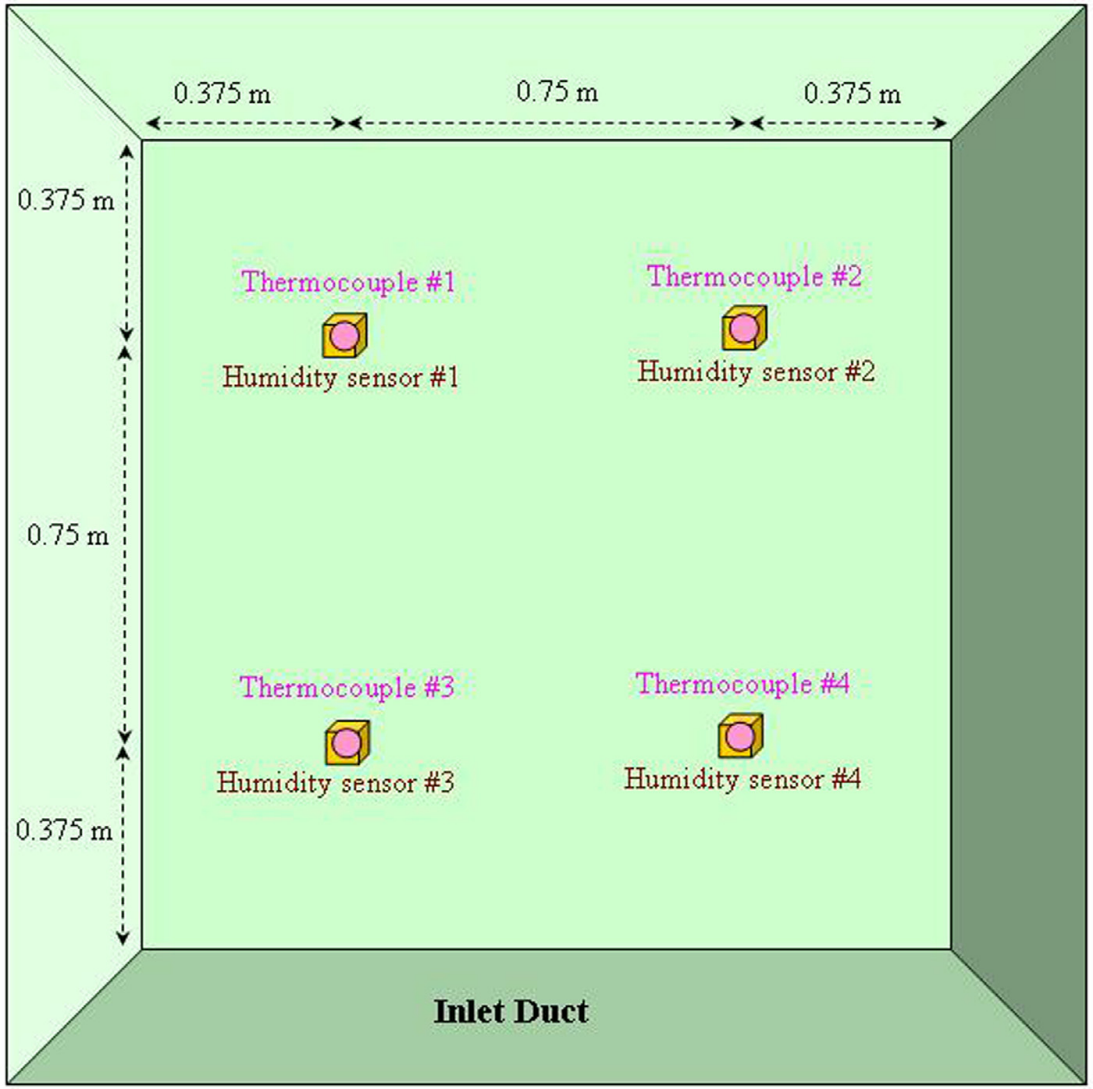
Temperature and humidity sensors proportionally spaced within the inlet duct.

The main conditions for collecting data are summarized as follows. Unconditioned outdoor air was introduced into the makeup inlet by an intake fan. Before mixing with the return air, inlet (ambient) temperature and humidity were measured via sensors behind the air filter. The placement of sensors is illustrated in Fig. 1 where four bundles of temperature/humidity sensors were proportionally spaced within the duct of 1.5 m by 1.5 m. Dry-bulb temperatures were measured through Type T (copper-constantan) thermocouples [2]. At the airflow side of the thermocouple is the junction of copper-constantan (four of them). At the data acquisition side of the thermocouple is the reference junction (copper-to-copper and constantan-to–copper) and into a plug-in HP 44421 A data acquisition board. All reference junctions were bunched together separately by electrical duct tapes in a well-insulated styrofoam box to ensure uniform temperature within the box. By ways of NIST defined 6^th^-order polynomials [3], all dc voltage readings were then converted into temperatures and stored into a computer adjacent to the air-handling unit that monitored and controlled the operation of the rotary wheel. Also stored into the data acquisition system were the humidity measurements [4] where chilled mirror hygrometers (D2 sensors) were employed.

**Fig. 2:**
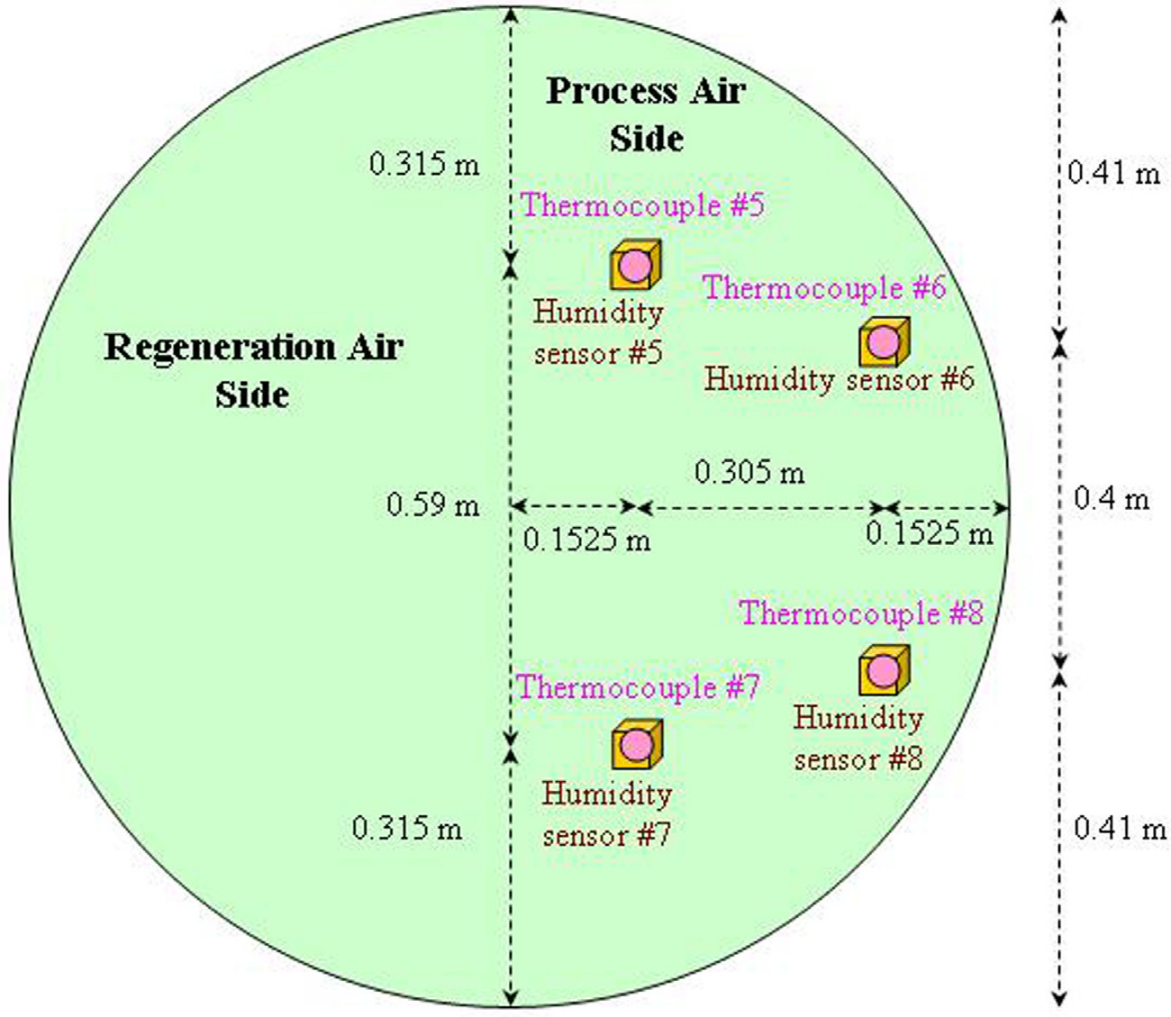
Temperature and humidity sensors proportionally spaced behind the rotary wheel.

After mixing of the outside air and the return air, the mixed air duct size (cross-section) was retrofitted (i.e. reduced) so that the mixed air would only flow through the process air side of the rotary desiccant wheel (i.e. half of the wheel) where the wheel diameter is 1.22 m and the wheel depth is 0.2 m (thickness) by Novel Aire Technologies. Four bundles of temperature/humidity sensors were also proportionally placed behind the process air side of the wheel (i.e. process air output), as shown in Fig. 2.

According to the manufacturer's static adsorption isotherm curves [5], the molecular sieve (LT3) curve exhibits higher moisture pickup at lower humidity and lower moisture pickup at higher humidity. The silica gel (WSG) curve exhibits lower desiccant capacities at lower humidity but rises to much higher desiccant capacities at higher humidity. Hence, silica gel (WSG) was the chosen sorption material of the rotary desiccant wheel over molecular sieve (LT3) because of the climate in Taiwan being mostly humid. Per ASHRAE [6], the particle radius of silica gel (WSG) is 0.0035 mm, with density at 1.129 g/m^3^. The specific heat (*c_p_*) of WSG is 1.82 kJ/kg-°C), compared with sea-level air (*c_p_* = 1.00 kJ/kg-°C) or water (*c_p_* = 4.186 kJ/kg-°C).

The experimental runs were performed to validate the simulation model. After validation of the model, optimal strategies of wheel operation would be determined from parametric analysis. As opposed to gradual changes, step changes were adapted to be consistent across the tests which actually represent the optimum or fastest possible change. Experimental validation would include the following experimental scenarios:

(1) Step increase and step decrease to the regeneration air temperature: A conventional desiccant system typically raises the temperature of the regeneration air stream flowing through the wheel for dehumidification. The facility uses the rejected heat from super-heated refrigerant as the heat source to superheat the regeneration air temperature via a heat exchanger before going through the cooling tower. Whenever a step change is applied to the regeneration air temperature, the reaction time is different for step increase and step decrease. Although not instantaneous, the step increase of the regeneration air temperature is much quicker than the step decrease of the regeneration air temperature. The time it takes for the desiccant wheel to return to a non-adsorbing steady-state condition is much longer whenever a step decrease of the regeneration air temperature is applied. The flow switching strategy would not work exactly in reverse for step increase and step decrease because the system must receive both process and regeneration air streams from different ambient conditions. Hence, step increase and step decrease to the regeneration air temperature were never conducted continuously. Step increase and step decrease of regeneration air temperature were done separately, by a few days apart.
(2) Step increase and step decrease to the wheel speed: The goal of step increase and step decrease to the wheel speed was to see significant effects to the transient response. This is the easiest part of the experiment because the motor that ran the wheel were direct. The wheel could be controlled to the desired speed instantaneously. For example, the tests may start from default at 18 rph, followed by a step increase to 36 rph, then a step decrease back to 18 rph. Or, the tests may start from the default at 18 rph, followed by a step decrease to 9 rph, then a step increase back 18 rph, as shown in the example data of Table 1.
(3) Step increase and step decrease to the process airflow rate: Because most cooling systems use variable-air-volume (VAV) control for ventilation, this is to simulate modulated airflow to cooling zones. The goal was to see the residual effect when the desiccant wheel encounters variations of airflow rates. Step increase of the process airflow rate would be compared to the step decrease of the process airflow rate. The tests would start from half of the designed process airflow rate at 400 fpm (2.032 m/s), follow by a step increase to the full designed process airflow rate at 800 fpm (4.064 m/s), then a step decrease back to 400 fpm (2.032 m/s). These variations of airflow rates were accomplished via dampers at the process air side.

## Ethics Statements

This work does not involve human subjects, animal experiments, or data collected from social media platforms.

## CRediT authorship contribution statement

**Hung-Yi Tsai:** Conceptualization, Methodology, Investigation, Supervision, Visualization, Software, Formal analysis. **Chung-Tai Wu:** Data curation, Validation, Writing – original draft, Writing – review & editing.

## Data Availability

In situ real-time dataset as compared to the predicted thermal wheel performance of an energy recovery system (Original data) (Mendeley Data).
